# A framework for assessing neuropsychiatric phenotypes by using smartphone-based location data

**DOI:** 10.1038/s41398-020-00893-4

**Published:** 2020-07-01

**Authors:** Niels Jongs, Raj Jagesar, Neeltje E. M. van Haren, Brenda W. J. H. Penninx, Lianne Reus, Pieter J. Visser, Nic J. A. van der Wee, Ina M. Koning, Celso Arango, Iris E. C. Sommer, Marinus J. C. Eijkemans, Jacob A. Vorstman, Martien J. Kas

**Affiliations:** 1 Groningen Institute for Evolutionary Life Sciences, University of Groningen, Groningen, The Netherlands; 2 Department of Child and Adolescent Psychiatry/Psychology, Erasmus Medical Center, Rotterdam, The Netherlands; 3 Department of Neuroscience, Department of Psychiatry, University Medical Center Groningen, University of Groningen, Groningen, The Netherlands; 4 Department of Psychiatry and Amsterdam Neuroscience, Amsterdam UMC, Vrije Universiteit, Amsterdam, The Netherlands; 5 Alzheimer Center Amsterdam, Department of Neurology, Amsterdam Neuroscience, Vrije Universiteit Amsterdam, Amsterdam UMC, Amsterdam, The Netherlands; 6 Department of Psychiatry, Leiden University Medical Center, Leiden, The Netherlands; 7 Leiden Institute for Brain and Cognition, Leiden University Medical Center, Leiden, The Netherlands; 8 Department of Social and Behavioural Sciences, Interdisciplinary Social Science, Youth Studies, Utrecht University, Utrecht, The Netherlands; 9 Institute of Psychiatry and Mental Health, Hospital General Universitario Gregorio Marañón, CIBERSAM, IiSGM, Universidad Complutense, School of Medicine, Madrid, Spain; 10 Julius Center for Health Sciences and Primary Care, Department of Biostatistics and Research Support, University Medical Center Utrecht, Utrecht, The Netherlands; 11 Department of Psychiatry, The Hospital for Sick Children and University of Toronto, Toronto, ON Canada; 12 Program in Genetics and Genome Biology, Research Institute, The Hospital for Sick Children, Toronto, ON Canada

**Keywords:** Neuroscience, Biomarkers

## Abstract

The use of smartphone-based location data to quantify behavior longitudinally and passively is rapidly gaining traction in neuropsychiatric research. However, a standardized and validated preprocessing framework for deriving behavioral phenotypes from smartphone-based location data is currently lacking. Here, we present a preprocessing framework consisting of methods that are validated in the context of geospatial data. This framework aims to generate context-enriched location data by identifying stationary, non-stationary, and recurrent stationary states in movement patterns. Subsequently, this context-enriched data is used to derive a series of behavioral phenotypes that are related to movement. By using smartphone-based location data collected from 245 subjects, including patients with schizophrenia, we show that the proposed framework is effective and accurate in generating context-enriched location data. This data was subsequently used to derive behavioral readouts that were sensitive in detecting behavioral nuances related to schizophrenia and aging, such as the time spent at home and the number of unique places visited. Overall, our results indicate that the proposed framework reliably preprocesses raw smartphone-based location data in such a manner that relevant behavioral phenotypes of interest can be derived.

## Introduction

The ability to objectively quantify different aspects of human behavior is essential for studies that aim to understand variations in human behavior and their underlying biological mechanisms. To date, such studies predominantly rely on subjective research methods such as in-person interviews, questionnaires and self- or proxy-rated measures. Subsequently, these behavioral phenotypic measures are used to examine interactions with an array of biological parameters, such as genotypes, brain activity patterns or structural brain data to study the biological underpinnings of the observed behavior. While such studies have led to numerous important insights, the current methods for behavioral phenotyping also have their limitations that preclude their objectivity. Most notably, these methods rely on the subject’s (or the subject’s proxy) account of behavior, and are invariably obtained post hoc, i.e. questionnaire measures of behavior are virtually never real-time. Observational assessments are real-time, but they occur nearly always in a non-natural (e.g., clinical) setting.

As a consequence, current behavioral assessment methods are susceptible to a wide variety of method and response biases^1^. These biases can cause systematic and random measurement errors^2^, thereby impeding the validity and interpretation of findings^3^. For example, when specific symptoms, such as cognitive dysfunction or lack of disease insight affect the subjective report of behavioral components, comparison between groups is severely hampered. Also, translational animal studies cannot use questionnaires, hence introducing additional divergence between animal and human assessments.

Recently, researchers have started to explore the utilization of smartphones as a more objective methodology to quantify human behavior^4^. Contemporary smartphones are equipped among others with sensors, such as a Global Positioning System (GPS), accelerometer, Bluetooth, Wi-Fi, microphone. These sensors can be used to collect a high-resolution trace of behavioral data, which can then be used to derive relevant behavioral markers. This method is increasingly referred to as “digital phenotyping”^5^ or “passive behavioral monitoring”^6^. Recent studies are already starting to reveal the clinical potential of the approach in the context of neuropsychiatric research^7–12^. The promise of this methodology is that the derived behavioral markers may provide unprecedented and unique insights into human behavior. Key features are (1) data is collected in real-time, 92) in the subject’s natural environment, and 93) without the need for any self- or proxy reporting, thereby addressing some of the most important challenges inherent to current behavioral research. Further adding to the appeal of using the smartphone is the relatively low-cost of this approach combined with the fact that the majority of people in western societies nowadays owns a smartphone^13^.

One of the most frequently used smartphone sensors in passive behavioral monitoring is the so-called GPS. The location data collected by this sensor primarily informs about the physical activity of participants but can also be used to explore different aspects of social behavior related to mobility. However, any single location data point in its raw state can only inform about the location in a two-dimensional space. In contrast, the analysis of multiple data points collected over time allows the inference of context. Contextualization, e.g., whether one is commuting between A and B, at home or visiting another location, is the basis for deriving relevant behavioral phenotypes from location data. This is acquired by a sequence of preprocessing procedures that enriches the raw location data. In addition, the process requires that a certain level of uncertainty in location data collected by smartphones is taken into account. Relevant behavioral phenotypes are subsequently derived by additional calculations on this context-enriched location data.

**Fig. 1:**
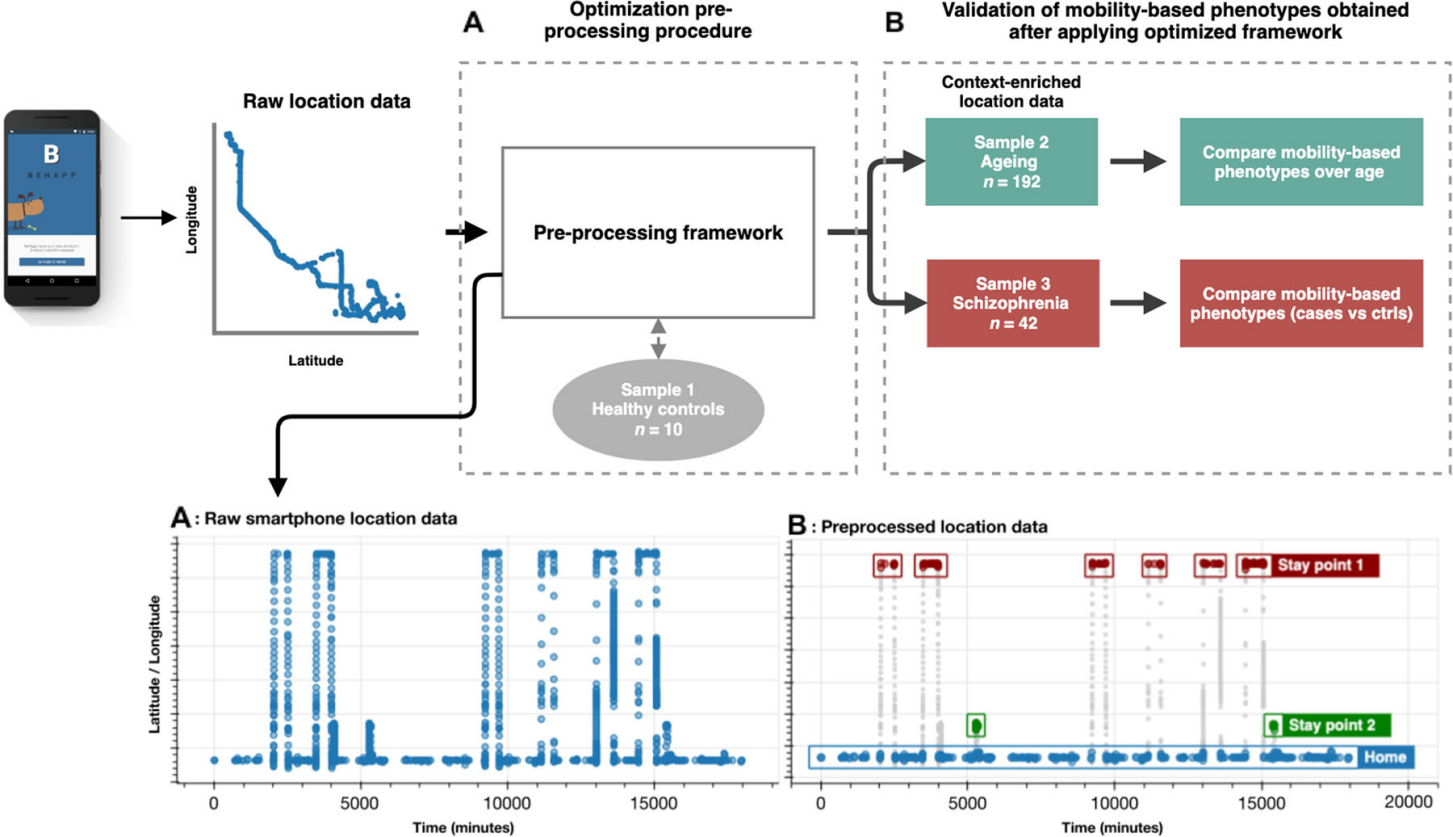
Overview experiments and utilization of the samples. **a** Sample 1 is used to develop and evaluate the preprocessing procedure used to contextualize raw location data collected by smartphones. **b** Samples 2 and 3 are used to validate the sensitivity of the derived behavioral readouts in detecting sample specific deviations of behavior. **c** Visualization of preprocessing procedure used to derive context-enriched location data. The raw location data (left figure) contains limited behavioral information and requires preprocessing to extract contextual information. Location data plotted over time combined with contextual information as derived by the preprocessing procedure (right figure). In this figure we can easily identify several stay points with repeated visits over time, the home location and travel patterns. This information is used to derive behavioral phenotypes such as the number of places visited (red and green), amount of home stay (blue) and the frequency of traveling (gray).

One major challenge in the utilization of smartphone-based location data for behavioral monitoring in research is the lack of a validated preprocessing framework to generate context-enriched location data. Previous studies that utilized smartphone-based location data^9–11,14–16^ to quantify behavior employed various generic preprocessing procedures that are not exclusively developed for location data. These methods are often not validated in context of location data and therefore often unable to deal with the uncertainty in location data. As a consequence, the use of these generic methods to preprocess location data might generate misleading behavioral phenotypes. In the present study, we (1) describe and evaluate the efficiency of a preprocessing procedure specifically developed for raw location data collected by smartphones, (2) describe a series of behavioral measures that are derived from these context-enriched location data, and (3) validate the sensitivity of the derived phenotypes in detecting behavioral nuances that are characteristic for specific populations samples (Fig. 1).

To achieve these goals, we collected location data by using a passive behavioral monitoring application called BEHAPP^6,17^ in three different samples. First, we collected data in a relatively small sample of healthy individuals (sample 1; *n* = 10) to optimize and evaluate the efficiency of the preprocessing procedures. Subsequently, these optimized preprocessing procedures were applied on data obtained in two additional samples (samples 2 and 3; *n* = 193 and *n* = 42 respectively) to generate context-enriched location data (Fig. 1c). The context-enriched location data from sample 2 and 3 was used to derive a set of six behavioral phenotypes that relate to several basic aspects of mobility and daily activities. Samples 2 and 3 were selected based on specific phenotypes (ageing and schizophrenia, respectively) known to be associated with certain behavioral characteristics, including relative changes in mobility patterns. Increasing age (sample 2) is characterized, for example, by a deterioration of the skeletal and muscular system which is known to impede mobility^18^. For this reason, we expected a relative decrease in mobility as a function of ageing. For schizophrenia (sample 3) it is known that the negative symptoms, which include decreased social engagement^19^ and initiative^20,21^, are associated with decreased mobility patterns^10^. Therefore, we expect mobility patterns to be affected in some of the derived phenotypes (e.g., time spent at home and visiting new places) for our sample of patients with schizophrenia relative to their age- and sex-matched controls.

In summary, we propose a preprocessing framework on smartphone-derived location data to allow contextualization and the inference of mobility-related phenotypes. Subsequently, we evaluated whether the smartphone-derived phenotypic measures of mobility were sufficiently sensitive to detect differences relative to controls and accordance with expectations given the impact of age or the presence of schizophrenia.

## Methods

### Participants

We recruited three different samples of participants to develop and evaluate the efficiency of the preprocessing framework (sample 1) and validate the sensitivity of the derived behavioral phenotypes (samples 2 and 3) in detecting sample-related behavioral changes (see Supplementary Materials and Supplementary Table 1 for sample specifications). The data in sample 2 and 3 was collected with approval from the concerned institutional ethics review boards and written informed consent was provided by al subjects.

### Data collection

The location (GPS) data used for this study was collected by the BEHAPP^22^ application (see Supplementary Materials for further details, including an example of geospatial coordinates collected by smartphones in supplementary table 2).

### Preprocessing procedures

The primary aim of our preprocessing procedure is to differentiate between stationary and non-stationary states, and cluster those stationary states that are recurrent over time. Examples of stationary states include being at home, visiting a relative or being at work. Movements within these stationery states are still considered stationary. Traveling from work to home or from work to a supermarket are examples of non-stationary states. We identified these stationary states by employing a stay point detection algorithm^23^ on raw location data that is filtered on accuracy. This stay point detection algorithm requires two parameters, a distance parameter $\theta _d$, and a time threshold parameter $\theta _t$. These threshold parameters $\theta _t$ and $\theta _d$ were fixed at 60 min for $\theta _t$ and 350 m for $\theta _d$. With these parameters, a single stay point is detected by the algorithm if a group of geospatial coordinates remains stationary for 60 min within an area of 350 m. A further description of the preprocessing procedures is provided in the Supplementary Materials.

### Smartphone-based behavioral phenotypes

In the Supplementary Materials we provide a full description of the behavioral phenotypes that are derived from the context-enriched location data. This context-enriched location data is extracted from the raw location data by using the optimized preprocessing procedure described above. The described phenotypes are proven to be sensitive in detecting behavioral nuances related to neuro-psychiatric disorders^9,11^.

### Statistical analysis groupwise comparisons

For sample 2 we used a one-way ANOVA with a Tukey post hoc test to study the association between the age bins and the derived behavioral phenotypes. In order to approach normality for the derived phenotypes we used a log transformation on those phenotypes that deviated from normality. Visual inspection of each phenotype was performed to assess normality.

For the SZ sample (sample 3) we performed a Poisson generalized linear model with a Tukey post hoc test to study the difference between the SZ and their controls on the number of stay points, trajectories and the number recurrent stay points. For the remaining phenotypes (percentage of home stay, normalized entropy and diurnal movement) we used a one-way ANOVA with a Tukey post hoc test to evaluate the difference between SZ and controls. For both sample 2 and 3 the assumptions of the used statistical methods were checked prior to analysis.

Prior to analyzing the phenotypes in sample 2 and 3 we adjusted the count-based phenotypes for the length of data collection by using the residuals of a linear model. Additionally, the effect of gender was also assessed for sample 1 and 2 on each phenotype by using a *t*-test and the results revealed a non-significant effect of gender and was therefore, not included in any further analysis.

## Results

### Preprocessing procedures (sample 1)

**Fig. 2:**
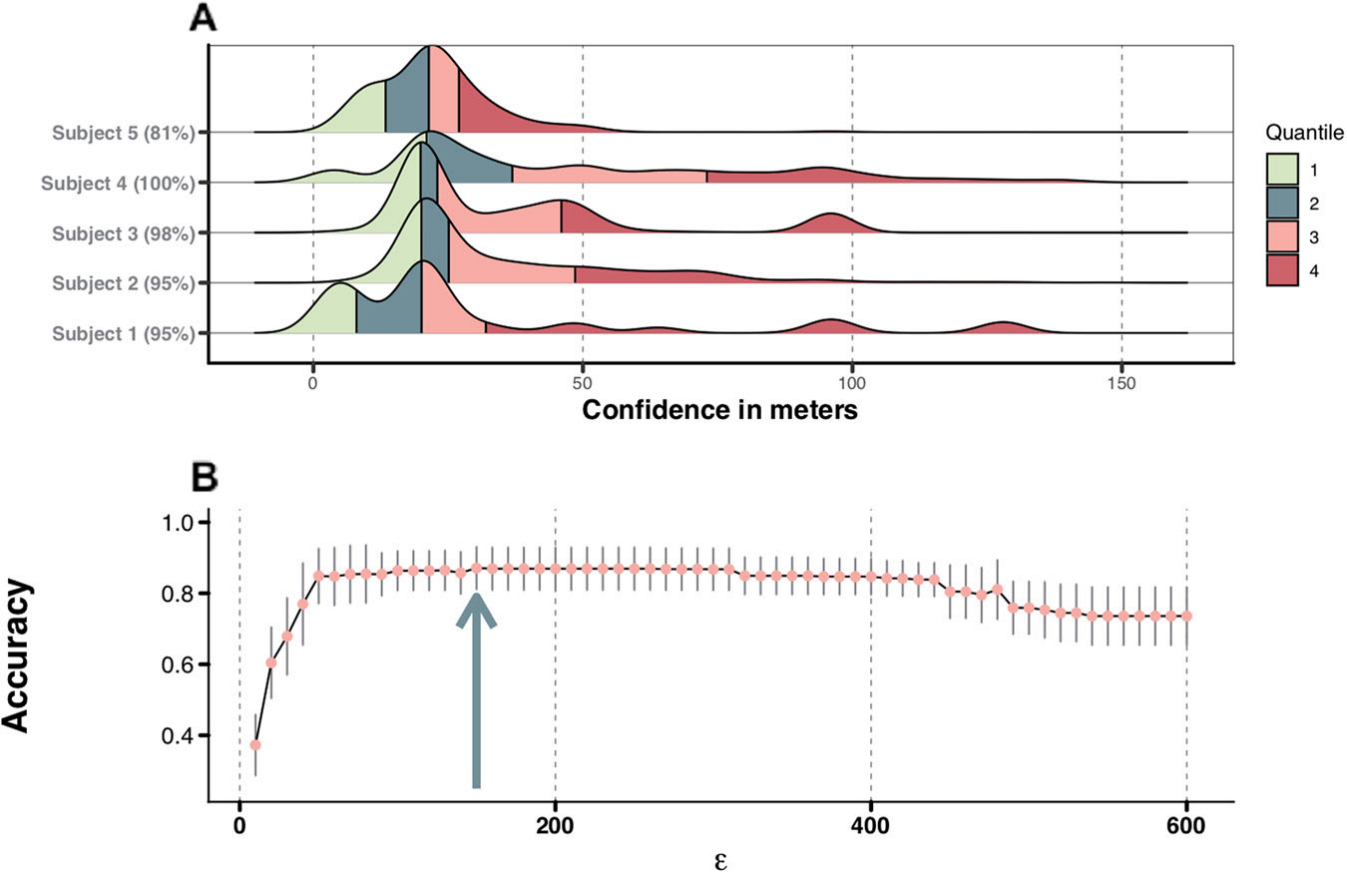
Evaluation and optimization preprocessing procedures. **a** Accuracy of the geospatial coordinates for each participant represented by the confidence in meters (colored in 25% quantiles). The confidence of each geospatial coordinate is interpreted as the 68% probability that true location is within the distance in meters. **b** Adjusted Rand Index for each epsilon value combined with the standard error. The optimal $\in$ value is 150m with an Adjusted Rand Index of 0.87, this point is marked by the arrowed line.

The preprocessing procedure serves (1) to enrich raw smartphone-based location data with contextual information by identifying non-stationary and stationary states and (2) to identify clusters of the latter that are recurrent over time. User confirmed validation of stationary states from five subjects collected over a period of 2 weeks showed that the accuracy (percentage correct) of the stay point detection algorithm^23^ in correctly identifying stationary states is 94%($\mu$) ± 8%(${\mathrm{sd}}$). The accuracy of the algorithm ranged between 100% and 95% for four out of five subjects (95%, 95%, 98%, 100%). The performance of the algorithm was substantially less for one subject with an accuracy of 81%. Closer inspection revealed that the collected location data from this subject was relatively more precise (Fig. 2a). The precision of geospatial coordinates is denoted by their confidence in meters. This confidence is interpreted as the 68% probability that the true location is within a specific range of proximity to the measured coordinates. The average confidence of this subject was with 24.9 m considerably lower than the 129.3, 108.9, 46.1, and 167.7 m that were observed for the remaining four subjects. Somewhat counter intuitively, the higher level of precision of GPS data in this subject led to a relatively lower accuracy of the stay point detection algorithm. These results suggest that the current definitions of the algorithm parameters (see “Methods”) tend to be more favorable on relative less precise GPS raw data.

The density-based clustering^24^ (DBSCAN) approach aimed at identifying recurrent stationary locations as a single entity. Our results revealed that by considering locations within a range of 150 m ($\in$ parameter; Fig. 2b) as a single entity, $87\%\;\pm\;13\%$ of the stationary locations with corresponding contextual meanings were correctly clustered together. This accuracy is defined as the percentage of stationary locations with corresponding contextual meaning correctly clustered together.

### Count-based phenotypes (samples 2 and 3)

Count-based behavioral phenotypes such as the number of places visited in total, unique places visited and trajectories, are directly derived from the preprocessed location data.

**Fig. 3:**
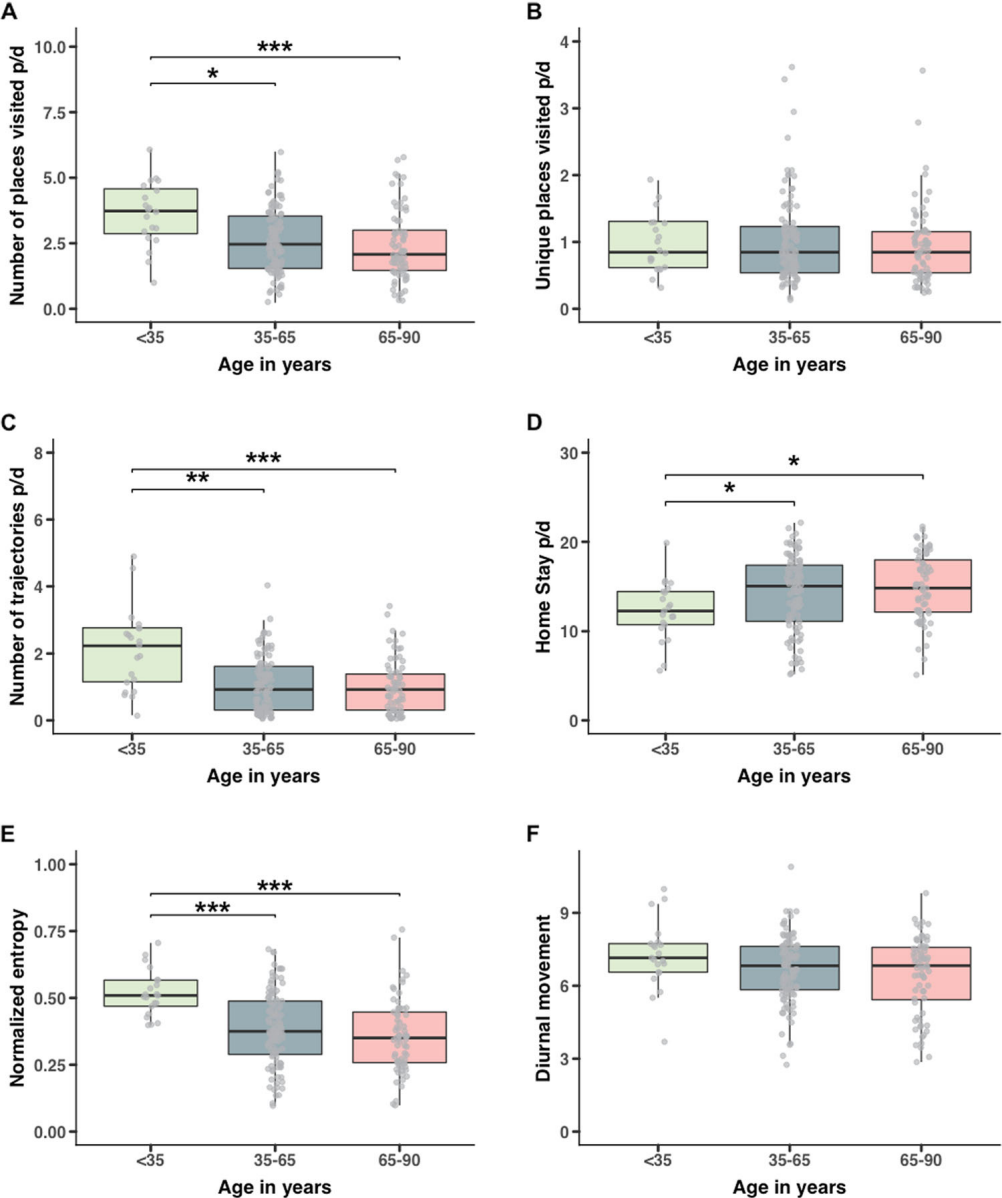
Behavioral phenotypes based on geospatial data for different age groupings. **a** Comparison of the number of places visited for three age bins showed that relative to the <35 the 35–65 (*p* = 0.012) and 65–90 (*p* < 0.001) group visited significant fewer places [*F*(2,190) = 7.14, *p* = 0.001]. **b** Number of unique places visited revealed non-significant differences for the three age bins [*F*(2,190) = 0.98, *p* = 0.378]. **c** Comparison of the number of trajectories revealed that number of trajectories was higher for the <35 relative to the 35–65 (*p* = 0.002) and 65–90 (*p* < 0.001) group [*F*(2,190) = 7.64, *p* < 0.001]. **d** Percentage of home stay is gradually and significantly increasing (*p* = 0.027, *p* = 0.017) with age [*F*(2,161) = 4.06, *p* = 0.019]. **e** Comparison of the normalized entropy measure revealed lower scores for the 35–65 (*p* < 0.001) and 65–90 (*p* < 0.001) group [*F*(2,190) = 12.03_,_*p* < 0.001]. **f** For the diurnal movement measure we did not find any significant differences. However, noteworthy is the difference in variance between the age groups which seems to increase by age [*F*(2,190) = 1.96, *p* = 0.144].

For sample 2 (ageing; *n* = 193), these count-based behavioral phenotypes revealed (Fig. 3a–c) significant differences between the middle aged (35–65 years) to elderly (65–90 years) subjects and the younger (<35 years) subjects. We found that relative to the younger group, middle aged and elderly subjects visited significant fewer places per day (young: 4.04 ± 2.26; middle: 2.76 ± 1.69; elderly: 2.41 ± 1.35) and traveled significant less on a daily basis (young: 2.10 ± 1.22; middle: 1.06 ± 0.84; elderly: 0.99 ± 0.82). With regard to the number of unique places visited, we found no difference between the elderly subjects and the other two age groups (young: 1.20 ± 1.13; middle: 1.02 ± 0.63; elderly: 0.94 ± 0.58).

**Fig. 4:**
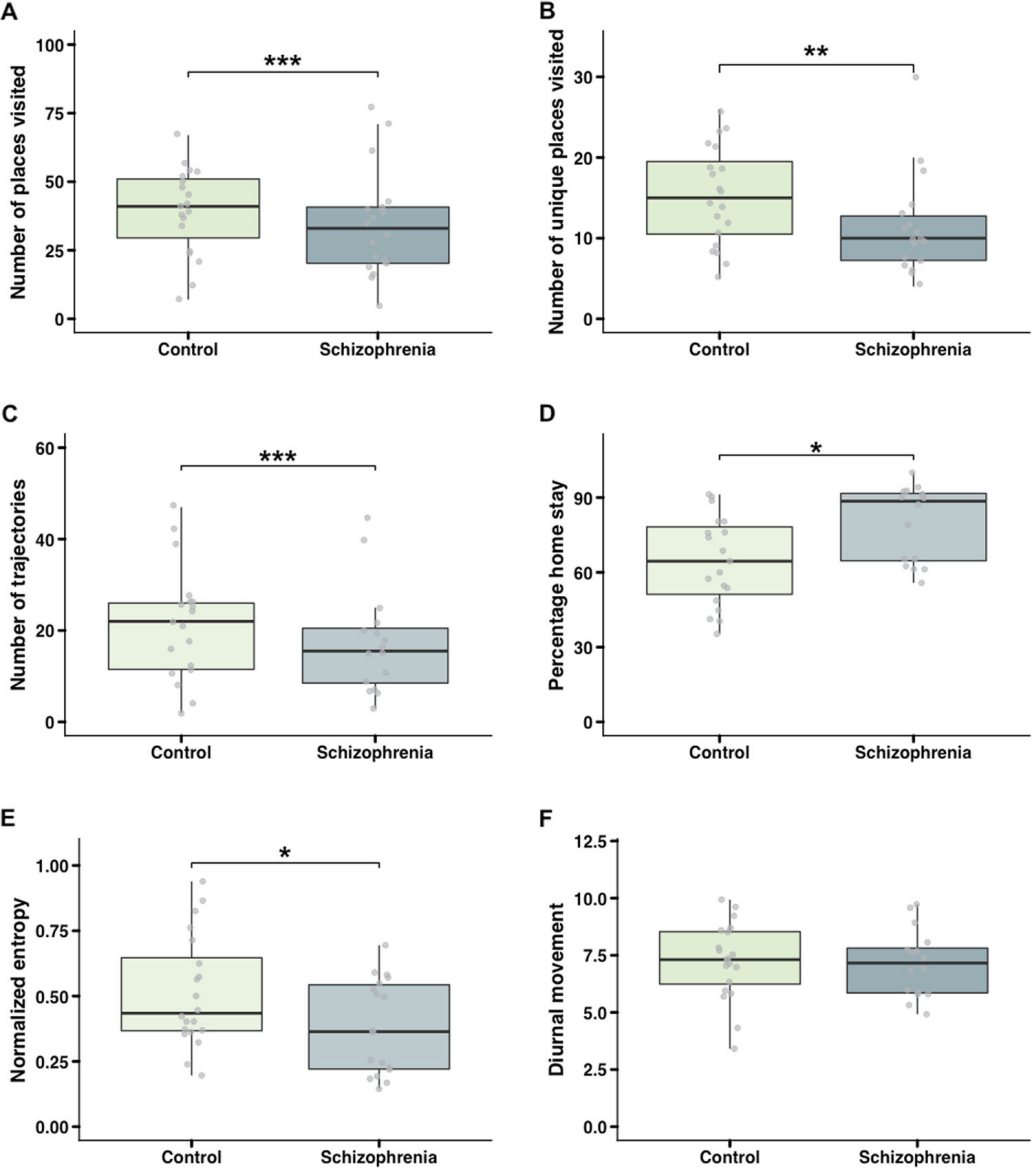
Behavioral phenotypes based on geospatial data for SZ and HC subjects. **a** Comparison of the number of places visited* for SZ and HC subjects showed that HC subjects visited significant more places [$\chi ^2\left( 1 \right)\,=\,18.813,\,p\,\leq\,0.001$]. **b** HC subjects visited significant more unique places* [$\chi ^2\left( 1 \right)\,=\,10.289,\,p\,=\,0.001$]. **c** Comparison of the number of trajectories* showed that HC subjects travel significant more than SZ subjects [$\chi ^2\left( 1 \right)\;=\,25.837,\,p\,<\,0.001$]. **d** Percentage of home stay reveal**e**d that SZ subjects spent significant more time at home compared to HC’s [$\chi ^2\left( 1 \right)\,=\,7.3878,\,p\,=\,0.006$]. **e** The results of the normalized entropy measure revealed that SZ subjects tend to spent significantly more time on a small set of stationary locations [$\chi ^2\left( 1 \right)\,=\,4.1058,\,p\,=\,0.04$]. **f** Comparison of the diurnal movement measure revealed a non-significant difference between SZ and HC subjects. (*Counts are adjusted for the number of days data collected).

For sample 3 (Schizophrenia (SZ); *n* = 42), these phenotypes showed that relative to the age- and sex-matched healthy control (HC) subjects the SZ subjects visited significant fewer places (HC: 43.35 ± 23.72; SZ 34.55 ± 19.44) (Fig. 4a). In addition to this, our results also showed that SZ subjects visited significant less unique places (HC: 15.25 ± 6.16*;* SZ: 11.44 ± 6.18; Fig. 4b) and traveled significant less often (HC: 25.25 ± 20.70; SZ: 15.00 ± 12.99; Fig. 4c).

### Home Stay (samples 1, 2, and 3)

To estimate the amount of home stay we used a heuristic-based rule (i.e. predefined rule) to identify the home location from a set of clustered stationary states as identified by the preprocessing procedure. We evaluated the accuracy of this heuristic-based rule by using the user confirmed clustered stationary states as provided by the subjects from sample 1. The results of this evaluation revealed that with an accuracy of 100% all home locations were correctly identified. Subsequently, we used this rule to infer the home location in sample 2 and 3, and subsequently, to determine the amount of home stay.

We used the amount of home stay per day to evaluate the association between home stay and increasing of age in sample 2. Our results revealed that the observed amount of home stay per day is significantly less in the younger subjects relative to the middle and elderly aged groups (young: 12.95 ± 2.67; middle: 15.15 ± 3.46; elderly: 15.25 ± 3.42; Fig. 3d). The observed amount of home stay between the middle (35–65) and elderly aged (65–90) did not differ significantly.

For sample 3, we used the same heuristic-based rule to estimate the percentage of home stay. Our results revealed that subjects diagnosed with SZ on average spent 15% (i.e. 3.6 h) more time at home as compared to HC’s (HC: 65% ± 18%; SZ: 80% ± 15%; Fig. 4a).

### Normalized entropy (samples 2 and 3)

**Fig. 5:**
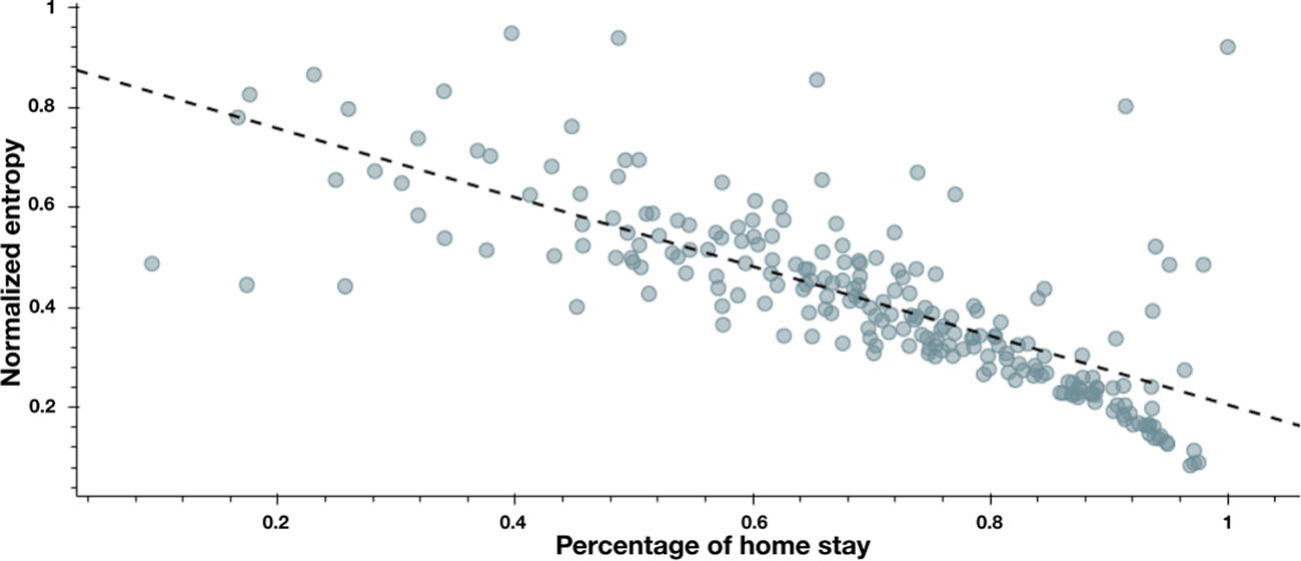
Normalized entropy measure plotted against the amount of home stay per day. The dashed line represents the linear model that was used to test the association between normalized entropy and the amount of home stay ($\beta = - 0.57, \, p \, < \, 0.001$). These results suggest that lower scores on the normalized entropy measure is correlated with increased home stay (*r*(191) = −0.72, *p* < 0.001).

Normalized entropy quantifies the variability of time spent at different stationary states^9,11,25^. Lower scores are observed on this measure when stay times are restricted to a small set of stationary states. Higher scores are observed when the time spent at different stationary states is more uniformly distributed across these stationary states. Given this definition, we found, as expected, a negative association between the normalized entropy measure and the percentage of home stay (Fig. 5, *r*(191) = −0.72, *p* < 0.001). This strong association is explained by the fact that spending more time at home leaves less time to visit other locations.

For the three age groups (sample 2), our results revealed significant differences relative to the younger subjects (<35). The results indicated greater inequality in the time spent across different stationary locations between the younger subjects and the middle and elderly aged subjects (Fig. 3e). For the younger group we observed an average normalized entropy of 0.52 ± 0.09 vs an average of 0.39 ± 0.14 and 0.36 ± 0.14 for the 35–60 and 60–90 groups, respectively.

The normalized entropy was also significantly different between SZ patients and age- and sex-matched HC’s (sample 3; Fig. 4e). It revealed a greater inequality in the time spent across different stationary locations for subjects diagnosed with SZ. The normalized entropy measure was on average 0.13 points higher in the HC group (HC: 0.51 ± 0.21; SZ: 0.38 ± 0.18).

### Diurnal movement (sample 2 and 3)

The regularity in movement patterns is measured by the diurnal movement phenotype^11^. Higher scores on this behavioral phenotype are observed in subjects with a repetitive and regular movement pattern within a 24-h period over a consecutive assessment of multiple days^11^ (including weekday and weekend). Results revealed that the regularity in movement patterns is similar for the three age groups (7.25 ± 1.40 vs 6.68 ± 1.38 vs 6.51 ± 1.69) (Fig. 3f).

We observed a small difference in the regularity of movement patterns between SZ and HC; (HC: 7.25 ± 1.67; SZ: 7.13 ± 1.47; Fig. 4f) respectively. However, this difference was not statistically significant.

## Discussion

The availability of objective and real-world behavioral phenotypes represents a fundamental change in our ability to study variation in human behavior. Here, we propose a framework to process raw smartphone collected geospatial data. We demonstrate that objective behavioral phenotypes of human behavior can be derived that are clinically relevant and are effective in detecting behavioral nuances consistent with expectation in specific population samples. This framework provides an important next step in the era of digital phenotyping, namely that of systematic pre-processing and validating passively monitored longitudinal raw data sets to deliver biologically relevant behavioral phenotypes of interest.

The sensitivity and usability of these behavioral phenotypes in detecting behavioral deviations is dependent on the efficiency of the preprocessing procedures. We demonstrate the efficiency of a two-step preprocessing procedure that utilizes a set of methods that are validated in the context of geospatial data^26,27^. Evaluation of this framework in terms of efficiency revealed an overall high accuracy in detecting stationary, non-stationary and recurrent stationary states correctly. We show that the stay point detection algorithm^23^ is able to detect stationary and non-stationary states with relatively high efficiency. Despite the relatively small size of sample 1 (*n* = 10), these results are in accordance with earlier findings that demonstrated the efficiency of this same algorithm in accurately detecting stationary and non-stationary states from smartphone-based location data^26,27^. Importantly, we found that the efficiency of the stay point detection algorithm is dependent on the interaction between the parameters as used by the algorithm and the precision of the collected geospatial coordinates. Our results suggest that under the current conditions the algorithm was less efficient on location data with relatively high precision. These findings indicated that the choice of these parameters should depend on the precision of the data that is used as input for the algorithm.

In addition, we used density-based clustering (DBSCAN^24^) with an optimized set of parameters to identify recurrent stationary states with identical entities. We showed that with these optimized parameters we were able to identify recurrent stationary states with high accuracy ($87\%$). This finding is consistent with results of earlier work that demonstrated the efficiency of the DBSCAN in clustering stationary states with identical entities together^28^. It is important to bear in mind that this approach remains relatively limited when it comes to differentiating between two distinct stationary states that are close in space. This limitation is due to the range parameters in DBSCAN that takes into account the variability in coordinates for stationary states with an identical entity. As a consequence, stationary states with distinct entities that are close in space are likely to be identified as a single entity due to the uncertainty that is introduced by this range parameter.

Overall, our results suggest that the proposed preprocessing steps (stay point detection and DBSCAN clustering) are efficient and reliable in detecting stationary and recurrent stationary states. Given that non-stationary states are defined as the inverse of stationary states, our results also provide evidence that non-stationary states are effectively identified by these preprocessing procedures.

We utilized these stationary and non-stationary states to formulate a set of behavioral phenotypes, which subsequently proved to be sensitive in detecting important behavioral nuances in our population samples. In the sample that included subjects across a wide age range (sample 2), our results revealed changes in these phenotypes that are likely associated with processes of aging. For example, relative to the younger subjects, we found that the middle and elderly aged subjects visited fewer places, traveled less and spent more time at home. Our findings did not reveal a difference between the middle and elderly aged subjects. We had expected a difference in mobility patterns, since the elderly group could be hypothesized to have a weaker fitness due to age-related physical changes in the skeletal and muscular system^18^ and elderly could be expected to be less active since they most often have retired from work. The difference between the results and these initial expectations are likely related to the fact that we did not take into account that the elderly group is likely to be more active due to retirement, while the context of work in the middle-aged group is likely associated with a higher frequency of sedentary lifestyle due to employment status. which is in accordance with earlier findings^29^. Arguably, one could still expect differences in certain aspects of mobility that were not captured by the phenotypic endpoints measured in the current study.

In addition, we found significant differences between SZ and HC subjects. For instance, we showed that subjects diagnosed with SZ significantly visited fewer unique places, traveled less and spent more time at home as compared to their age- and gender-matched controls. These significant differences may be indicators of reduced social behavior and may relate to the known diminished social functioning in the SZ group^20,30^ and other psychiatric disorders^31^. These findings are comparable to an earlier location data based phenotype of decreased exploratory behavior in patients with depression who are also known to suffer from social withdrawal^9,11^. Alternatively, these findings could also be driven by a different attitude of patients with schizophrenia towards smartphones (e.g., averse due to paranoid tendencies) or cognitive impairments that cause patients to leave their smartphone at home. Therefore, while our findings are consistent with what is known about social behavior in schizophrenia, alternative explanations exist which are unrelated to the social functioning of a patient. Additional studies addressing parallel social functioning and smartphone monitoring assessments are needed to extend the validation of digital measures of social behavior in these patient cohorts.

It is important to emphasize that we used age and neuropsychiatric disease status to demonstrate that the location-based derived behavioral phenotypes are sufficiently sensitive to detect behavioral nuances characteristic to specific populations. While our results evidently demonstrate this sensitivity, it also suggests the importance of taking into account demographical factors when using these phenotypes for groupwise comparisons. Demographical factors such as age, employment status, living in a rural or urban area, or disability status have the potential to affect the derived phenotypes. For example, employment status and living in a rural area might affect the distance travelled and factors such as age have an effect on the number of places visited and the amount of time spent at home as showed in this paper. Without the availability of subjects matched on the basis of several demographical factors, the ability to compare these phenotypes between different populations/groups is limited and might lead to wrong conclusions.

It is also noteworthy that the interpretation of the derived phenotypes is limited to the definition of how the stationary locations are identified here. While we have shown that the stay point detection algorithm^23^ as used here is accurate in detecting stationary locations, the movement within stationary locations (i.e. buildings) is not registered by this approach. With regard to the derived phenotypes this indicates that the movement within stationary locations is not taken into account by the derived phenotypes. This restricts the behavioral interpretation of these phenotypes and is therefore, limited to the definition of a stationary location. This inability to detect movement within stationary locations is due to the constraint that smartphone-based location data solely reflects movement with a degree of uncertainty and provides a rough estimation of the true location. Additional smartphone sensors such as the accelerometer could potentially be utilized to quantify movement with stationary locations and enrich the information used to derive phenotypes.

We expect that passive monitoring strategies have an important potential for both research and clinical care related to human behavior and mental health. For research, implementation of these methods will generate behavioral data that is unlike any of the currently existing data in this field in terms of their objective nature, their high resolution and their acquisition in a natural, real world setting. There is also clinical potential; we speculate that objective measures of mobility can provide, at least theoretically, clinically relevant insights in a patient’s physical exercise and may also be related to their level of social engagement. Accuracy of the latter may be improved by combining GPS data with other data retrievable from smartphones related to communication (e.g. frequency of phone calls or texting). We identify two important next directions for further research towards validation of these potential clinical applications of passive monitoring strategies.

First, it is important to note that our findings for schizophrenia do not necessarily extrapolate to other psychiatric disorders. While we hypothesize that passive monitoring strategies will likely generate relevant insights for all psychiatric disorders, we expect that both nature and effect size of changes in mobility and social behavior patterns may reveal a combination of differentiating and overlapping signatures across disorders^32^. Therefore, an important next step in this field will be to validate this strategy for all major psychiatric disorders, and to evaluate differences and similarities between the observed behavioral patterns.

Second, for a clinical application it will be vital to also explore the extent to which changes in individual passive monitoring data patterns may be used to identify transitions in mental health status; for instance someone recovering from a depressive mood episode may be showing a gradual increase in mobility. Another example may be the detection of decrease in social interaction through passive monitoring a possible early warning signal for an impending recurrent psychotic episode in an individual diagnosed with schizophrenia.

In sum, we propose a framework to derive digital quantitative measures of human mobility that can be assessed in a longitudinal and objective manner in the real-world environment. Following preprocessing raw smartphone location data, human behavioral phenotypes have been developed, validated through user confirmation, and successfully applied to assess the effects of ageing and schizophrenia on these measures. We suggest that provided data is adequately processed, digital phenotyping has the potential to provide a new entry into the quantitative and more objective assessment of behavior in humans, allowing to expand our knowledge of the biological mechanisms that drive these behaviors. For neuropsychiatric disorders, this is the first step towards a scalable and more objective measure of behavior, which will be a critical step forward to improve our understanding of mental illness.

## Supplementary information

Supplementary materials
