# Supplementary material for: A framework for assessing neuropsychiatric phenotypes by using smartphone-based location data
Source: Transl Psychiatry. 2020 Jul 1;10:211. doi: 10.1038/s41398-020-00893-4 (PMC7329884; doi:10.1038/s41398-020-00893-4)
Supplement: Supplementary file 1 — Supplementary materials [file 41398_2020_893_MOESM1_ESM.docx]

Supplementary materials

# Participants:

**Sample 1:** Data from 10 healthy participants was used to evaluate the efficiency of the preprocessing framework used to generate context-enriched location data. These participants (6 males; 4 females) with an average age of 33.2 ± 13.74 verbally agreed to collect location data over a period of 2 weeks (14 days). After the data collection period, participants were asked to manually confirm the output of the preprocessing procedures. We obtained a complete and detailed confirmation of output in 5 out of 10 participants. The remaining 5 participants provided confirmation that was limited to the home location, as identified by using their smartphone data.

**Sample 2:** We recruited 193 healthy participants without any neurological or psychiatric condition that might affect the data collection or the derived phenotypes. We used these participants to validate the sensitivity of the behavioral phenotypes in detecting age specific differences. This group (81 males; 112 females) with an average age of 57.48 ± 14.71 years agreed to collect location data for 14 days with their own smartphone. Age was arbitrarily binned in three categories, namely <35 years (n = 21), 35 to 65 years (n = 103) and 65 to 90 years (n = 69) years to account for variations in the behavioral phenotypes. The greater part of the subjects in the 65 to 90 bin were recruited through hersenonderzoek.nl ([www.hersenonderzoek.nl](http://www.hersenonderzoek.nl)). These participants provided self-assessed information regarding the presence of a psychiatric condition and cognitive impairments. The remaining participants were screened on the absence of any neurological disease or psychiatric condition affecting the CNS, which is associated with cognitive impairment.

**Sample 3:** We recruited 21 individuals with a clinically confirmed diagnosis of schizophrenia (SZ) and 21 age-and gender matched healthy controls (HC). Sample 3 was used to assess the sensitivity of the behavioral phenotypes in detecting differences in social behavior between those with and without schizophrenia. Patients with SZ and controls were recruited as part of the ongoing Psychiatric Ratings using Intermediate Stratified Markers (PRISM) project^1^ and the Social Cognition and Imaging in Psychiatry (SCIP) study. SZ diagnoses were confirmed during recruitment by using the Comprehensive Assessment of Symptoms and History^2^ (CASH) questionnaire. The average age of this group (34 males, 8 females) was 31.78 ± 8.91. All participants provided written informed consent to collect at least 14 days of location data. Data collection in 3 out of the 42 participants failed due to smartphone compatibility problems.

*Supplementary Table 1| Overview of the characteristics per group and in which part of the study the data was used.*

|  | ***n*** | **Age** | **Gender** | | **Data collection period** | **Development preprocessing**  **procedures** | | **Validation**  **phenotypes** |
| --- | --- | --- | --- | --- | --- | --- | --- | --- |
| **Group 1** | 10 | 33.2 (*sd* = 13.74) | 6 M; 4 F | 13 days | | $\surd$ |  | |
| **Group 2** | 192 | 55.47 (sd = 14.71) | 81 M; 112 F | 13 days | |  | $\surd$ | |
| **Group 3** | 42 | 31.78 (sd = 8.91) | 34 M; 8 F | 14 or 42 days | |  | $\surd$ | |

# Data collection

BEHAPP is a passive behavioral monitoring application for Android that collects data by utilizing the embedded sensors in participants own smartphones. BEHAPP is used for scientific research that aims to provide objective, quantitative and longitudinal measures of human (social) behavior to classify mental health disorders based on digital behavioral profiles, develop digital biomarkers to study disease progression and treatment efficacy, and identify early indicators of disease that allow prediction of disease onset, relapse and remission.

The location data collected by BEHAPP for a single participant is defined as a four-dimensional matrix $C$ and holds the latitude and longitude, their corresponding time-stamps and accuracy indicators of the coordinates. Therefore, a single geospatial coordinate $i$ is defined as $C_{i}=\{{lat}_{i}, {lng}_{i},t_{i,}{acc}_{i}\}$ where $i=1,2, ...,p$. The accuracy of smartphone-based location data is rarely 100% exact and is irregular over time due to sensor noise and environmental factors. The accuracy of a coordinate is described in terms of their confidence in meters. This confidence is interpreted as the 68% probability that the true location is within the radius of the circle around the observed coordinate. Coordinates that exceeded an accuracy of 350 meters were excluded from further computations. An example of smartphone-based location data is given in supplementary Table 2, in this example the third record accommodates the most accurately observed coordinate.

***Supplementary Table 2 |*** *Example of geospatial coordinates collected by smartphones. The latitude and longitude provide information about the actual location, time provides some contextual and temporal information about this actual location. The accuracy values inform about the reliability of the observed location. In this example the third observation is the most reliable in terms of accuracy.*

| **Latitude** | **Longitude** | **Time** | **Accuracy(m)** |
| --- | --- | --- | --- |
| 53.24149 | 6.537214 | 14:49:15 | 48.000 |
| 53.24086 | 6.537754 | 15:00:26 | 128.000 |
| 53.24136 | 6.537314 | 15:00:47 | 20.006 |
| 53.24118 | 6.536491 | 15:56:58 | 48.000 |
| 53.24088 | 6.536467 | 15:57:00 | 32.000 |
| 53.24081 | 6.536918 | 15:57:16 | 32.000 |

# Preprocessing procedures

A set of $v$ stationary states for a single subject is denoted as $S = \left\{ s_{1}, s_{2}, ...,s_{v} \right\},$ where a distinct stationary state is defined as $s_{j} = \{t_{j}^{a}, t_{j}^{d},{lat}_{j}, {lng}_{j}\}.$ Here, $t_{j}^{a}$ and $t_{j}^{d}$ contain the arrival and departure time for stationary state $j$, ${lat}_{j}$ and ${lng}_{j}$ hold the coordinates for stationary state $j$. The coordinates for stationary state $j$ are obtained by taking the average of the latitude and longitude of the coordinates that belong to stationary state $j$. The efficiency of the stay point detection algorithm in correctly identifying stationary states was evaluated with the afore mentioned parameters by analyzing the user confirmed output of the algorithm. User confirmed output was provided by 5 of the 10 participants from sample 1 (supplementary Table 1).

Recurrent stationary states over time are clustered by using a density-based clustering^3^ (DBSCAN) approach which groups stationary states that are close in space. Two stationary states are considered close in space if directly reachable by a pre-specified distance. DBSCAN requires two parameters; a minimum number of points needed to establish a cluster $(MinPts= 2)$, and an $\epsilon$ parameter. The $\epsilon$ parameter defines the maximum space of the neighborhood for a single stationary location and is crucial for clustering locations. A new cluster is established if two stay points are directly reachable by the distance as defined by ϵ. This distance between stationary states is defined by the haversine distance function which calculates the distance between a pair of coordinates denoted in latitude and longitude. By using this distance function, a distance matrix $D$ of size $v \times v$ with pairwise distances between all observed stationary states was generated and used as input for the DBSCAN algorithm. The output of this approach was used to assign each stationary state to a cluster of recurrent stationary locations. These clusters of recurrent stationary locations are denoted by $cl=\{{cl}_{1},{cl}_{2},,\ldots,{cl}_{j}\}$ where ${cl}_{j}$ is the cluster index for stationary location $j$.

We used the data collected in sample 1 to perform an optimization experiment to find the optimal $\epsilon$ value that maximizes the performance of DBSCAN. For a range of $\epsilon$ values between 10 and 600 meters the accuracy (Adjusted Rand Index) of DBSCAN was assessed by comparing the user grouped stationary locations with the clustered stationary states. User grouped stationary locations were again provided by 5 of the 10 subjects from the sample 1. The $\epsilon$ value with the highest accuracy was chosen as the final parameter for DBSCAN and was used to derive behavioral phenotypes from the preprocessed location data.

Non-stationary states are identified by using the inverse of the output as generated by the stay point detection algorithm. This approach defines all the geospatial coordinates between two consecutive stationary states as non-stationary when a set of conditions is satisfied. We used a heuristic-based rule to exclude trajectories that do not exceed a minimum of 20 coordinates. We consider it unlikely, that trajectories with less than 20 coordinates are a good representation of the transition between two consecutive stay points. A set of trajectories is denoted as $TR = \{{tr}_{1},{tr}_{2},..., {tr}_{n_{tr}}\}$ and contains $n_{tr}$ trajectories. A single trajectory is denoted as $tr = \{t_{j}^{d}, t_{j+1}^{a},{COR}_{tr}\}$ where $t_{j}^{d}$ denotes the departure time at stay point $j$and $t_{j+1}^{a},$ the arrival time at the consecutive stay point $j+1.$The corresponding set of geospatial coordinates for a single trajectory is denoted as ${COR}_{tr}= C[t_{j}^{d} t_{j}^{a}].$ The accuracy of this approach is depended on the performance of the stay point detection algorithm as described above So, a highly accurate stay point detection algorithm directly translates into accurate trajectories.

# Smartphone-based behavioral phenotypes

*Count-based phenotypes.* Smartphone-based behavioral phenotypes such as the number of places visited, number of unique places visited, and the number of trajectories are directly derived from the context-enriched location data. So, with the definitions given above, the number of places visited is equal to $v$, the number of unique places visited is equal to the unique values in $cl$ and the number of trajectories is equal to $n_{tr}$.

For sample 3, these count-based phenotypes are adjusted for the length of the data collection period by using simple linear regression. This linear model is fitted by using:

$$\hat{y}= \beta_{0}+ \beta_{1}x_{period}$$

where $y$ is the count-based phenotype and $x_{period}$ the length of the data collection period. The adjusted phenotype is then defined as the residuals added to the average of the count-based phenotype:

$$\bar{y}+\left( \hat{y}-y \right).$$

*Home Stay.* Home stay is defined as the total amount of time observed at home during the data collection period. For this behavioral phenotype, it is crucial to correctly identify those geospatial coordinates that reside with the actual home location $H$. In order to achieve this, we defined a heuristic-based rule that estimates the home location from a set of clustered stationary locations (see preprocessing procedures). This heuristic-based rule estimates the home location by selecting those three clustered stationary locations with the most hours observed at night (between 00:00 AM and 06:00 AM). Subsequently, the most frequently visited stationary location of these three is considered the home location $H$.

The total amount of home stay $HS$ is defined by the sum of the hours observed at the estimated home location $H$,

$$HS= \sum_{j=cl=H}^{1} \left( t_{j}^{d}- t_{j}^{a} \right),$$

and measures the total time observed at home. The percentage of home stay is defined by the amount home stay divided by the sum of the total time spent at stationary locations:

$HSP= \frac{HS}{\sum_{j=}^{1} \left( t_{j}^{d}- t_{j}^{a} \right)}$ .

This percentage of home stay is favorable over the total amount of home stay if the length of the data collection period varies between subjects such as in the SZ sample (sample 3).

We evaluated this heuristic rule by comparing the home locations provided by the 10 subjects from group 1 with the estimated home locations. The accuracy of this rule in estimating the correct home location is expressed in percentage correctly estimated home locations. For the groupwise comparisons in sample 2 and 3, observations with less than 35% (8.4 hours a day) of home stay were removed from further analysis since it is unlikely that the derived amount of home stay is calculated by the using the correct home location.

*Normalized entropy.* The normalized entropy^4^ measure is calculated by using the percentage of time spent at different clusters of stationary locations and is defined by:

$ENT= \frac{-\sum_{l} p_{l} \log_{10}(p_{l})}{\log_{10}(N_{cl})}$.

where each $l = 1,2, ...,N_{cl}$ refers to a clustered set of stationary locations, and where $N_{cl}$ is the observed number of clusters. $p_{l}$ denotes the percentage of time spent at location cluster $l$. This readout measures the degree of variability in stay time over the clustered stationary locations. Higher normalized entropy values indicate more uniformly distributed stay times across clusters and lower values are observed when the stay times are focused around a single cluster. Because of this, the normalized entropy is expected to be negatively associated with the percentage of home stay.

*Diurnal movement.* Diurnal movement (DM) measures the regularity in daily movement patterns. Subjects with stable daily movement patterns score higher on this phenotype compared to subjects with irregular daily movement patterns^5,6^. The DM measure is calculated by using the Lomb-Scargle periodogram^7^ . The Lomb-Scargle periodogram is used to determine the power within a 24 ± 0.5-hour cycle for the distance between the home location $H$ and the observed stationary locations. The distance between the home location $H$ and a single stationary location $j$ is again defined by haversine distance function. The power spectral density (PSD) of these distances for a 24 ± 0.5-hour period is estimated and averaged by the $K$ frequencies in the 24 ± 0.5 cycle by applying

$$P_{d}=\sum_{t}^{K} {psd(f_{t})}/K .$$

Where $psd(f_{t})$ is the $PSD$of the data at time bin $t$ that is within a 24 ± 0.5-hour period bin.

The power $P_{d}$ is calculated for the normalized distances between the home location and the stationary locations and is considered the final measure of DM. By using these normalized distances, the DM measure becomes insensitive to outliers and the distance traveled^6^.

# References:

1. Kas, M. J. *et al.* A quantitative approach to neuropsychiatry: The why and the how. *Neurosci. Biobehav. Rev.* **97**, 3–9 (2019).

2. Andreasen, N. C., Flaum, M. & Arndt, S. The Comprehensive Assessment of Symptoms and History (CASH): An Instrument for Assessing Diagnosis and Psychopathology. *Arch. Gen. Psychiatry* (1992). doi:10.1001/archpsyc.1992.01820080023004

3. Ester, M., Kriegel, H. P., Sander, J. & Xu, X. A Density-Based Algorithm for Discovering Clusters in Large Spatial Databases with Noise. *Proc. 2nd Int. Conf. Knowl. Discov. Data Min.* (1996). doi:10.1.1.71.1980

4. Shenkin, P. S., Erman, B. & Mastrandrea, L. D. Information‐theoretical entropy as a measure of sequence variability. *Proteins Struct. Funct. Bioinforma.* (1991). doi:10.1002/prot.340110408

5. Saeb, S. *et al.* Mobile phone sensor correlates of depressive symptom severity in daily-life behavior: An exploratory study. *J. Med. Internet Res.* **17**, 1–11 (2015).

6. Palmius, N. *et al.* Detecting Bipolar Depression from Geographic Location Data. 1–10 (2015).

7. Van Dongen, H. P. A., Olofsen, E., VanHartevelt, J. H. & Kruyt, E. W. Searching for biological rhythms: Peak detection in the periodogram of unequally spaced data. in *Journal of Biological Rhythms* (1999). doi:10.1177/074873099129000984
